# Pre-sarcopenia determines post-progression outcomes in advanced hepatocellular carcinoma after sorafenib failure

**DOI:** 10.1038/s41598-020-75198-z

**Published:** 2020-10-27

**Authors:** Tsung-Yi Cheng, Pei-Chang Lee, Yi-Tzen Chen, Yee Chao, Ming-Chih Hou, Yi-Hsiang Huang

**Affiliations:** 1 Division of Gastroenterology and Hepatology, Department of Medicine, Taipei Veterans General Hospital, Taipei, Taiwan; 2 Department of Nursing, Taipei Veterans General Hospital, Taipei, Taiwan; 3 Department of Oncology, Taipei Veterans General Hospital, Taipei, Taiwan; 4 Institute of Pharmacology, National Yang-Ming University School of Medicine, Taipei, Taiwan; 5 Institute of Clinical Medicine, National Yang-Ming University School of Medicine, Taipei, Taiwan; 6 Faculty of Medicine, National Yang-Ming University School of Medicine, Taipei, Taiwan

**Keywords:** Malnutrition, Risk factors, Liver cancer

## Abstract

Many second-line therapies are recently approved for patients with advanced hepatocellular carcinoma (HCC), in whom protein malnutrition is prevalent that would affect treatment outcomes. In this study, we aimed to investigate the role of pre-sarcopenia and muscle restoration in patients with sorafenib-failed advanced HCC. From August 2012 to March 2017, 385 patients who developed radiology-proven HCC progression after sorafenib treatment were enrolled in the study. Pre-sarcopenia is defined as transverse psoas muscle thickness per body height < 16.8 mm/m, which was prevalent (64.7%) in our patients. Age > 60 years, female gender, and body mass index < 22 kg/m^2^ were independent predictors to the development of pre-sarcopenia. Patients with muscle depletion had significantly worse post-progression survival (PPS) compared with their counterparts (median PPS: 3.8 vs. 5.8 months, *p* = 0.003), particularly in those with intermediate liver reserves (Child–Pugh class B or Albumin-bilirubin grade 2). Besides, pre-sarcopenia independently predicted post-progression mortality in sorafenib-failed HCC (hazard ratio: 1.340, *p* = 0.012). In patients who developed pre-sarcopenia before sorafenib treatment, muscle restoration was associated with a longer PPS compared with their counterparts (6.3 vs. 3.6 months, *p* = 0.043). In conclusion, pre-sarcopenia independently determined the outcomes of sorafenib-failed HCC. Nutrition support to restore muscle mass would prolong survival for higher-risk patients.

## Introduction

Hepatocellular carcinoma (HCC) ranks as the sixth most common cancer and the fourth cause of cancer-related deaths worldwide, with increased incidence to nearly 750,000 new cases per year^1,2^. In general, the prognosis of HCC is relatively dismal because only 46% of patients can be diagnosed at an early stage, and most patients require systemic therapy for the unresectable, advanced-staged disease^2,3^. Sorafenib is the first approved systemic treatment for advanced HCC; but its effects are still modest^4,5^. Recently, several positive results from the first and second-line phase 3 trials enable HCC patients to new treatment options^6–8^. Maintenance of good liver reserve and performance status at disease progression, which are generally measured by Child–Pugh class, ALBI grade or Eastern Cooperative Oncology Group (ECOG) status, are important for longer survival with second-line therapy^2,6,9,10^.

Depletion of muscle and decline of muscle strength are common in patients with advanced liver disease and malignancies, which are reported in association with cachexia and indicated as prognostic factors for them^11–15^. A significant proportion of patients with HCC were observed as malnourished or at a high risk of malnutrition, which remarkably affected clinical outcomes^16^. In parallel with liver dysfunction and enlargement of tumor size, skeletal muscle decreases profoundly in patients with advance HCC^17^. Presence of muscle depletion can lead to physical disability and is associated with poor prognosis in these patients^18–20^. According to previous studies, pre-sarcopenia, which is defined as low skeletal muscle mass assessed by computed tomography (CT) scan, can predict the prognosis of HCC in patients treated with sorafenib^18–23^. However, whether this objective, quantitative surrogate marker of performance status can predict post-progression prognosis in sorafenib-failed patients is still unclear. In addition, factors linked to pre-sarcopenia must be identified. In this study, we aimed to investigate the role of muscle mass depletion in HCC patients who failed sorafenib treatment as well as the risk factors for the presence of pre-sarcopenia in these patients.

## Results

### Demographic characteristics of the study cohort

According to the value of transverse psoas muscle thickness per body height (TPMT/BH), which was measured from the CT scan image at the level of umbilicus, 249 patients (64.7%) showed pre-sarcopenia at the time of sorafenib treatment failure. Compared with patients with normal muscle mass, patients with the presence of pre-sarcopenia at HCC progression were significantly older (patients with pre-sarcopenia vs. patients with normal muscle mass: 64.0 ± 12.4 vs. 61.4 ± 13.5 years old, *p* = 0.029), predominantly female (25.7% vs. 14.0%, *p* = 0.009), lower in body mass index (BMI) (23.0 ± 3.8 vs. 25.0 ± 4.1, *p* < 0.001), more prevalent in chronic hepatitis C infection (30.1% vs. 16.9%, *p* = 0.005), with larger tumor size (6.6 vs. 5.5 cm, *p* = 0.015), and associated with shorter duration of sorafenib treatment (median treatment duration: 67 vs. 80 days, *p* = 0.026).

Regarding functional liver reserve, presence of pre-sarcopenia was significantly associated with prolonged prothrombin time (international normalized ratio [INR]: 1.15 vs. 1.09, *p* = 0.001), lower serum albumin level (3.2 vs. 3.4 g/dL, *p* = 0.011), and a higher incidence of ascites formation (49.2% vs. 39.7%, *p* = 0.018). After sorafenib treatment failure, most patients received the best supportive care from August 2012 to March 2017. No significant difference in post-progression treatment was identified according to the patients’ muscle status. Detailed characteristics of patients with progressive disease (PD) of HCC after sorafenib treatment are presented in Table 1.

**Table 1:** Characteristics of 385 PD patients classified by the presence or absence of pre-sarcopenia.

Characteristics at PD	Pre-sarcopenia	Normal muscle mass	*p* value
	n = 249	n = 136	
Age, years	64.0 ± 12.4	61.4 ± 13.5	0.029
Sex (female), n (%)	64 (25.7)	19 (14.0)	0.009
BMI, kg/m^2^	23.0 ± 3.8	25.0 ± 4.1	< 0.001
Underlying disease, n (%)			
Chronic hepatitis B	153 (61.4)	101 (74.3)	0.013
Chronic hepatitis C	75 (30.1)	23 (16.9)	0.005
Alcoholic liver disease	15 (6.0)	8 (5.9)	1.000
Tumor max size at PD, cm	6.6 (3.8–12.0)	5.5 (3.1–9.4)	0.015
Tumor numbers (single/multiple), n (%)	24/225 (9.6/90.4)	14/122 (10.3/89.7)	0.837
Sorafenib treatment duration, day	67 (55–128)	80 (58–151)	0.026
Reduced 75% sorafenib dose	102 (41.0)	68 (50.0)	0.107
Early PD within 4 months, n (%)	197 (79.1)	101 (74.3)	0.277
Lab data at PD			
AFP, ng/mL	945.9 (42.6–177,744.9)	972.7 (45.5–12,511.5)	0.853
TPMT/BH, mm/m	13.2 (10.7–14.8)	19.4 (18.1–21.2)	< 0.001
Prothrombin time, INR	1.15 (1.07–1.28)	1.09 (1.02–1.21)	0.001
Platelet count, K/cumm	140 (92–215)	132 (100–208)	0.921
Creatinine, mg/dL	0.82 (0.69–0.98)	0.84 (0.73–1.00)	0.271
ALT, U/L	45 (27–67)	47 (28–75)	0.849
AST, U/L	69 (47–135)	65 (41–108)	0.097
Total bilirubin, mg/dL	1.27 (0.79–2.15)	1.26 (0.73–2.10)	0.416
Albumin, g/dL	3.2 (2.8–3.7)	3.4 (2.9–3.9)	0.011
Ascites (none/mild/severe), n (%)	126/111/11 (50.8/44.8/4.4)	82/54/0 (60.3/39.7/0)	0.018
Child–Pugh class A/B/C	119/112/18 (47.8/45/7.2)	80/47/9 (58.8/34.6/6.6)	0.109
ALBI grade 1/2/3, n (%)	33/151/65 (13.3/60.6/26.1)	27/83/26 (19.9/61/19.1)	0.118
Progression pattern, n (%)			
New intrahepatic metastasis	93 (37.3)	55 (40.4)	0.584
New extrahepatic metastasis	108 (43.4)	54 (39.7)	0.518
Intrahepatic growth	108 (43.4)	57 (41.9)	0.830
Extrahepatic growth	67 (26.9)	35 (25.7)	0.904
Progressive vascular invasion	112 (45.0)	61 (44.9)	1.000
Extrahepatic metastasis	179 (71.9)	99 (72.8)	0.906
Major vascular invasion	164 (65.9)	88 (64.7)	0.823
Post Sorafenib treatment			
Chemotherapy	17 (6.8)	17 (12.5)	0.089
Radiotherapy	38 (15.3)	25 (18.4)	0.472
Immunotherapy	8 (3.2)	5 (3.7)	0.776
Best supportive care	195 (78.3)	95 (69.9)	0.083

*AFP* alpha fetoprotein, *ALBI grade* albumin-bilirubin grade, *ALT* alanine aminotransferase, *AST* aspartate aminotransferase, *BMI* body mass index, *ECOG* Eastern Cooperative Oncology Group, *INR* international normalized ratio, *Mets* metastasis, *MVI* macrovascular invasion, *PD* progressive disease, *TPMT/BH* transverse psoas muscle thickness per body height.

### Factors associated with pre-sarcopenia in sorafenib-failed HCC

In Table 2, age > 60 years (odds ratio [OR]: 1.796; 95% CI 1.126–2.863, *p* = 0.014), female gender (OR: 1.877; 95% CI 1.774–3.220, *p* = 0.045), and BMI < 22 kg/m^2^ (OR: 4.116; 95% CI 2.216–7.646, *p* < 0.001) were independent predictors of pre-sarcopenia in sorafenib-failed HCC by multivariate analysis.

**Table 2:** Factors associated with pre-sarcopenia in sorafenib-failed HCC.

	Univariate			Multivariate		
	OR	95% CI	*p*	OR	95% CI	*p*
Age, years						
> 60 vs. ≦60	1.626	1.063–2.488	0.025	1.796	1.126–2.863	0.014
Sex						
Female vs. Male	2.130	1.214–3.737	0.008	1.877	1.774–3.220	0.045
BMI, kg/m^2^ &						
BMI ≧ 22.0, ascites (−)	1	–	–	1	–	–
Ascites status						
BMI ≧ 22.0, ascites ( +)	1.327	0.815–2.159	0.255			NS
BMI < 22.0	4.089	2.287–7.310	< 0.001	4.116	2.216–7.646	< 0.001
Chronic viral hepatitis						
Yes vs. No	1.175	0.615–2.242	0.626			NA
Alcoholism						
Yes vs. No	1.026	0.423–2.484	0.955			NA
Lab data at PD						
AFP, ng/mL						
> 400 vs. ≦ 400	0.749	0.483–1.163	0.198			NA
Creatinine, mg/dL						
> 1.2 vs.+ 1.2	1.675	0.857–3.273	0.131			NA
Prothrombin time, INR						
> 1.2 vs.≦ 1.2	1.630	1.019–2.609	0.042			NS
ALT, U/L						
> 40 vs.≦ 40	1.168	0.767–1.780	0.469			NA
AST, U/L						
> 40 vs.≦ 40	1.282	0.762–2.155	0.350			NA
Platelet count						
≦ 100 K vs. > 100 K	1.252	0.772–2.030	0.362			NA
ALBI grade at PD						
Grade 2 vs. 1	1.488	0.838–2.645	0.175			NS
Grade 3 vs. 1	2.045	1.034–4.047	0.040			NS
Tumor size, cm						
> 7 vs. ≦ 7	1.381	0.904–2.109	0.135			NA
Tumor number						
Multiple vs. Single	0.973	0.638–1.483	0.899			NA
Early progression*						
Yes vs. No	1.313	0.803–2.145	0.277			NA
Extrahepatic metastasis						
Yes vs. No	0.956	0.599–1.526	0.849			NA
Major vascular invasion						
Yes vs. No	1.052	0.679–1.632	0.819			NA

*AFP* alpha fetoprotein, *ALBI grade* albumin-bilirubin grade, *ALT* alanine aminotransferase, *AST* aspartate aminotransferase, *BMI* body mass index, *CI* confidence interval, *INR* international normalized ratio, *NA* not adopted, *NS* not significant, *OR* odds ratio, *PD* progressive disease.

*Early progression: progressive disease developed within 4 months during sorafenib treatment.

**Figure 1:**
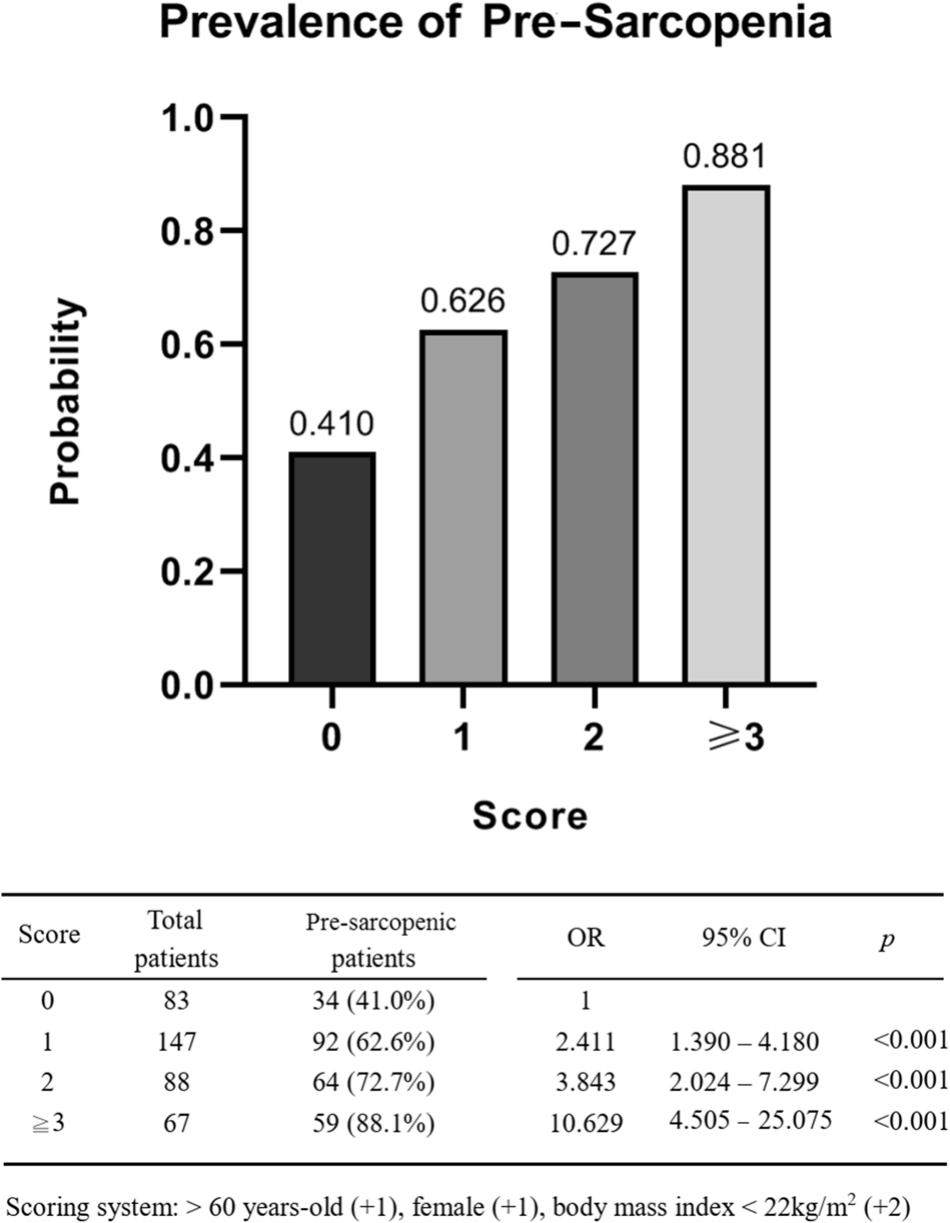
A scoring system to predict pre-sarcopenia in sorafenib failed HCC. Scoring system: > 60 years-old (+ 1), female (+ 1), body mass index < 22 kg/m^2^ (+ 2).

According to the beta coefficient values in multivariate analysis, a scoring system was established to predict the presence of pre-sarcopenia in sorafenib-failed HCC by incorporating gender, age, and BMI at HCC progression. Patients with scores > 0 had a significantly higher risk of pre-sarcopenia (*p* < 0.001). In addition, the risk and prevalence of pre-sarcopenia increased in alignment with these scores (Fig. 1).

### Post-progression survival (PPS) Associated with Pre-sarcopenia

**Figure 2:**
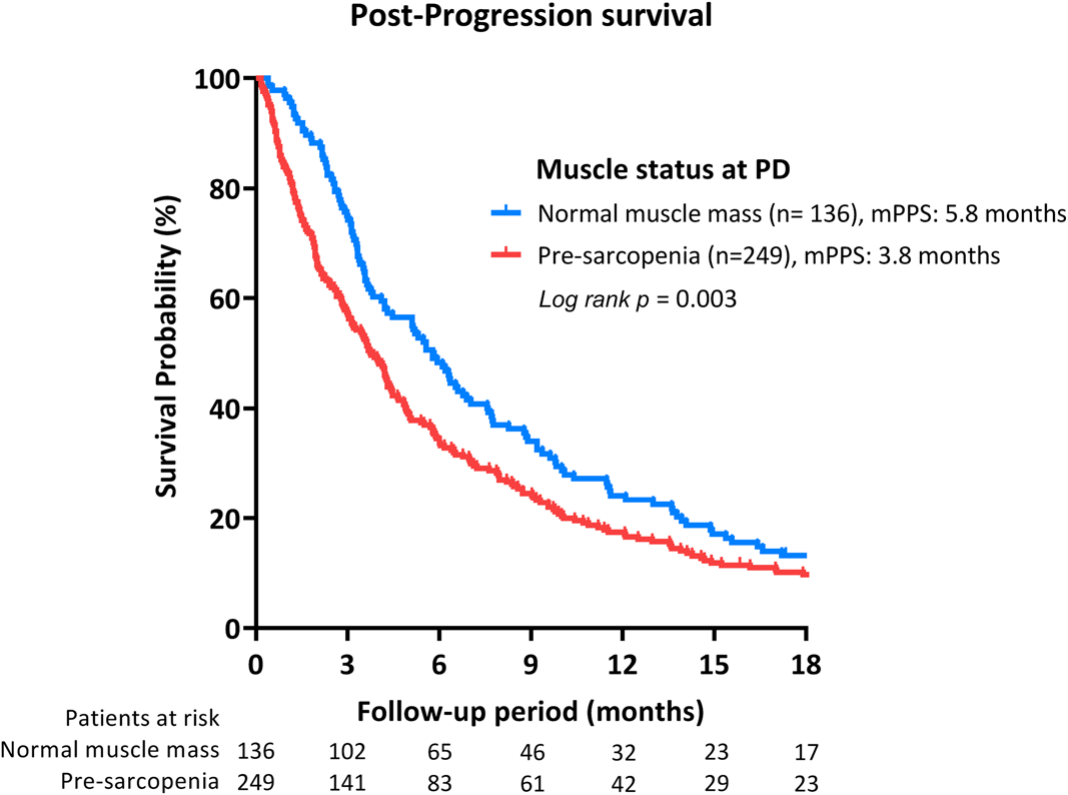
Pre-sarcopenia discriminates post-progression survival (PPS) of sorafenib-failed, advanced hepatocellular carcinoma. Abbreviation: PD, progressive disease.

Following progression of 4.2 months (interquartile range: 1.9–9.6), 358 deaths occurred with the median PPS as 4.2 months (95% CI: 3.6–4.9). Patients presented with pre-sarcopenia while HCC progression had significantly worse PPS than others without remarkable muscle depletion (median PPS: 3.8 vs. 5.8 months, *p* = 0.003) (Fig. 2). Among patients who had normal muscle mass before sorafenib treatment (n = 194), the presence of pre-sarcopenia at PD suggested a shorter PPS than muscle maintainers (4.1 vs. 5.6 months, *p* = 0.112). In patients with the presence of pre-sarcopenia before sorafenib treatment (n = 191), reversal of pre-sarcopenia while tumor progression was significantly associated with a longer PPS compared with their counterparts (6.3 vs. 3.6 months, *p* = 0.043) (Supplementary Fig. 1).

**Figure 3:**
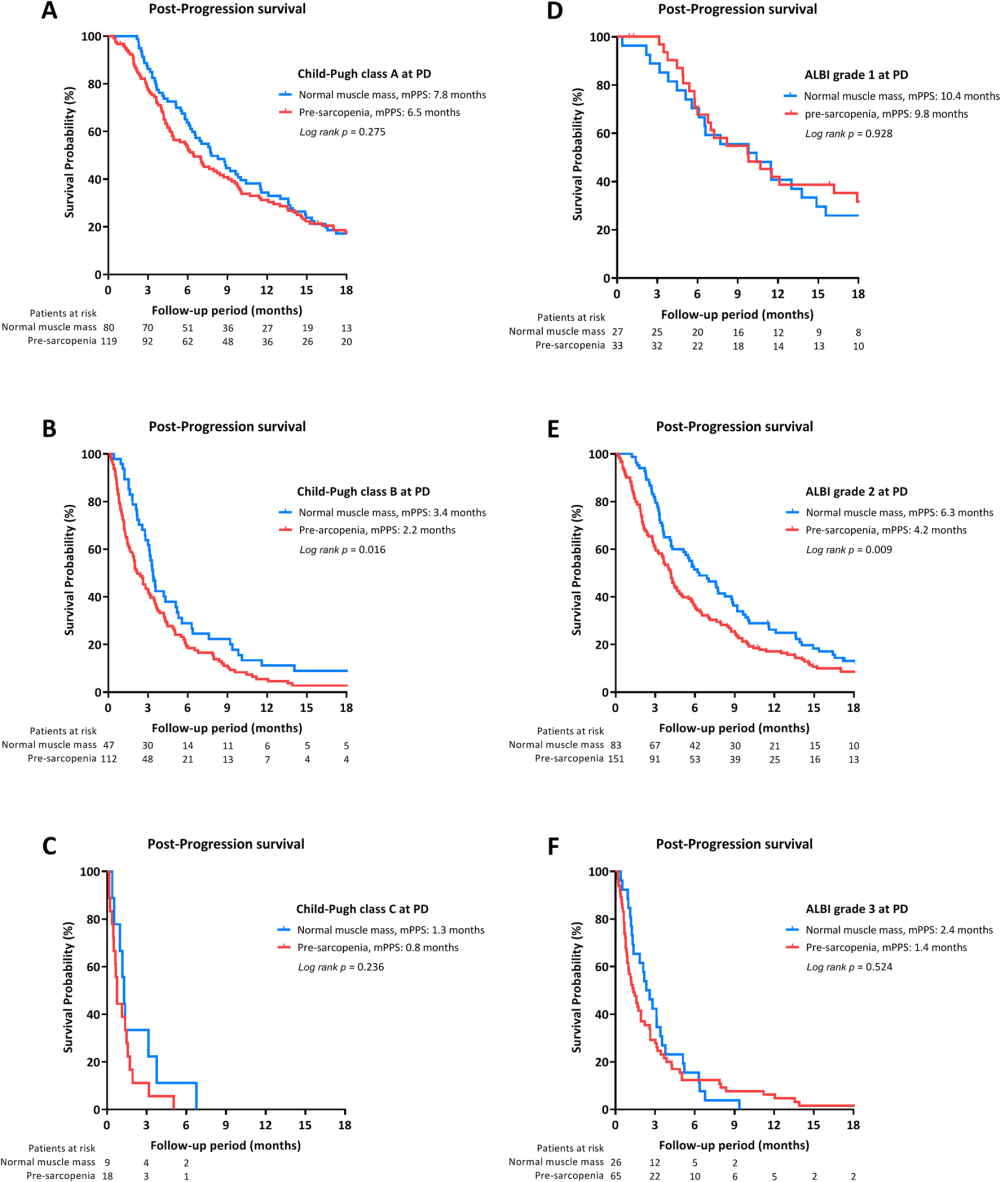
Subgroup analysis of post-progression survival (PPS) according pre-sarcopenia. Subgroup analysis of PPS based on the presence of sarcopenia among patients at Child–Pugh class A–C (**A**–**C**) and ALBI grade 1–3 (**D**–**F**).

As shown in Fig. 3A–C, the presence of pre-sarcopenia at PD significantly determined PPS in patients at Child–Pugh class B while HCC progression (3.4 vs. 2.2 months, *p* = 0.016). However, PPS was not significantly different based on muscle status in patients at Child–Pugh A or C. Similar results were also noted by evaluating liver function according to the albumin-bilirubin (ALBI) grade. The PPS was significantly better in patients with ALBI grade 2 with normal muscle mass versus those with the presence of pre-sarcopenia (6.3 vs. 4.2 months, *p* = 0.009). Nevertheless, the muscle-dependent survival difference was not observed in patients at ALBI grade 1 or 3 while tumor progression (Fig. 3D–F).

### Prognostic factors associated with PPS

Independent factors associated with PPS were determined by multivariate analysis. To avoid the effect of collinearity, Child–Pugh class and ALBI grade were not included in the same multivariate model. For the analysis of model 1 (Table 3), presence of pre-sarcopenia at PD (hazard ratio [HR]: 1.404; 95% CI 1.112–1.773, *p* = 0.004), maximal tumor size > 7 cm (HR: 1.722; 95% CI 1.356–2.186, *p* < 0.001), serum level of alpha-fetoprotein (AFP) > 400 ng/mL (HR: 1.322; 95% CI 1.044–1.673, *p* = 0.020), Child–Pugh class C (HR: 5.429; 95% CI 3.351–8.706, *p* < 0.001), early PD within 4 months of sorafenib treatment (HR: 1.388; 95% CI 1.058–1.822, *p* = 0.018), and the presence of new extrahepatic metastasis (HR: 1.783; 95% CI 1.414–2.248, *p* < 0.001) were independent risk factors for worse PPS in patients who failed sorafenib treatment for advanced HCC. In model 2 of multivariate analysis, ALBI grade 3 (HR: 4.209; 95% CI 2.864–6.186, *p* < 0.001), presence of pre-sarcopenia, larger tumor size, higher AFP, early PD, and new extrahepatic metastasis, were independent survival predictors.

**Table 3:** Factors associated with post-progression survival in sorafenib-failed HCC.

	Univariate			Multivariate (model 1)^#^			Multivariate (model 2)^#^		
	HR	95% CI	*p* value	HR	95% CI	*p* value	HR	95% CI	*p* value
Age, years									
> 60 vs. ≦ 60	1.008	0.880–1.346	0.435			NA			NA
Sex									
Male vs. Female	1.024	0.795–1.318	0.856			NA			NA
BMI	0.970	0.942–0.998	0.035			NS			NS
HBV infection									
Yes vs. No	1.059	0.850–1.320	0.609			NA			NA
HCV infection									
Yes vs. No	0.797	0.626–1.013	0.064			NA			NA
Alcoholism									
Yes vs. No	1.197	0.777–1.843	0.415			NA			NA
Pre-sarcopenia at PD									
Yes vs. No	1.390	1.116–1.730	0.003	1.404	1.112–1.773	0.004	1.340	1.065–1.685	0.012
Tumor size, cm									
> 7 vs. ≦ 7	2.233	1.800–2.769	< 0.001	1.722	1.356–2.186	< 0.001	1.797	1.417–2.279	< 0.001
Tumor number									
Multiple vs. Single	0.770	0.550–1.079	0.129			NA			NA
Lab data at PD									
AFP, ng/mL									
> 400 vs. ≦ 400	1.599	1.278–2.001	< 0.001	1.322	1.044–1.673	0.020	1.363	1.080–1.722	0.009
Creatinine, mg/dL									
> 1.2 vs. ≦ 1.2	1.698	1.254–2.300	0.001			NS			NS
Prothrombin time, INR									
> 1.2 vs. ≦ 1.2	2.473	1.969–3.107	< 0.001			NS			NS
ALT, U/L									
> 40 vs. ≦ 40	1.095	0.859–1.306	0.590			NA			NA
AST, U/L									
> 40 vs. ≦ 40	1.378	1.057–1.796	0.018			NS			NS
Platelet count									
< 100 K vs. ≧ 100 K	0.798	0.628–1.013	0.064			NS			NS
ALBI grade at PD									
Grade 2 vs. 1	1.817	1.333–2.476	< 0.001			NA			NS
Grade 3 vs. 1	4.872	3.405–6.970	< 0.001			NA	4.209	2.864–6.186	< 0.001
Child–Pugh class at PD									
Class B vs. A	2.354	1.885–2.940	< 0.001			NS			NA
Class C vs. A	7.636	4.981–11.705	< 0.001	5.429	3.351–8.706	< 0.001			NA
Early progression*									
Yes vs. No	1.569	1.212–2.031	0.001	1.388	1.058–1.822	0.018	1.324	1.007–1.740	0.044
Progression pattern									
New intrahepatic mets									
Yes vs. No	1.071	0.865—1.325	0.531			NA			NA
New extrahepatic mets									
Yes vs. No	1.793	1.448–2.219	< 0.001	1.783	1.414–2.248	< 0.001	1.889	1.499–2.380	< 0.001
Intrahepatic growth									
Yes vs. No	1.526	1.236–1.884	< 0.001			NS			NS
Extrahepatic growth									
Yes vs. No	0.892	0.705–1.127	0.338			NA			NA
Progressive vascular invasion									
Yes vs. No	1.390	1.128–1.712	0.002			NS			NS

AFP, alpha fetoprotein; ALBI grade, albumin-bilirubin grade; ALT, alanine aminotransferase; AST, aspartate aminotransferase; CI, confidence interval; HBV, hepatitis B; HCV, hepatitis C; HR, hazard ratio; INR, international normalized ratio; Mets, metastasis; NA, not adopted; NS, not significant; PD, progressive disease.

*Early progression: progressive disease developed within 4 months during sorafenib treatment.

^#^Model 1 enrolled parameters with *p* value < 0.2 in univariate analysis into multivariate analysis, except ALBI grade.

^#^Model 2 enrolled parameters with *p* value < 0.2 in univariate analysis into multivariate analysis, except Child–Pugh class.

#### Subgroup analysis

**Figure 4:**
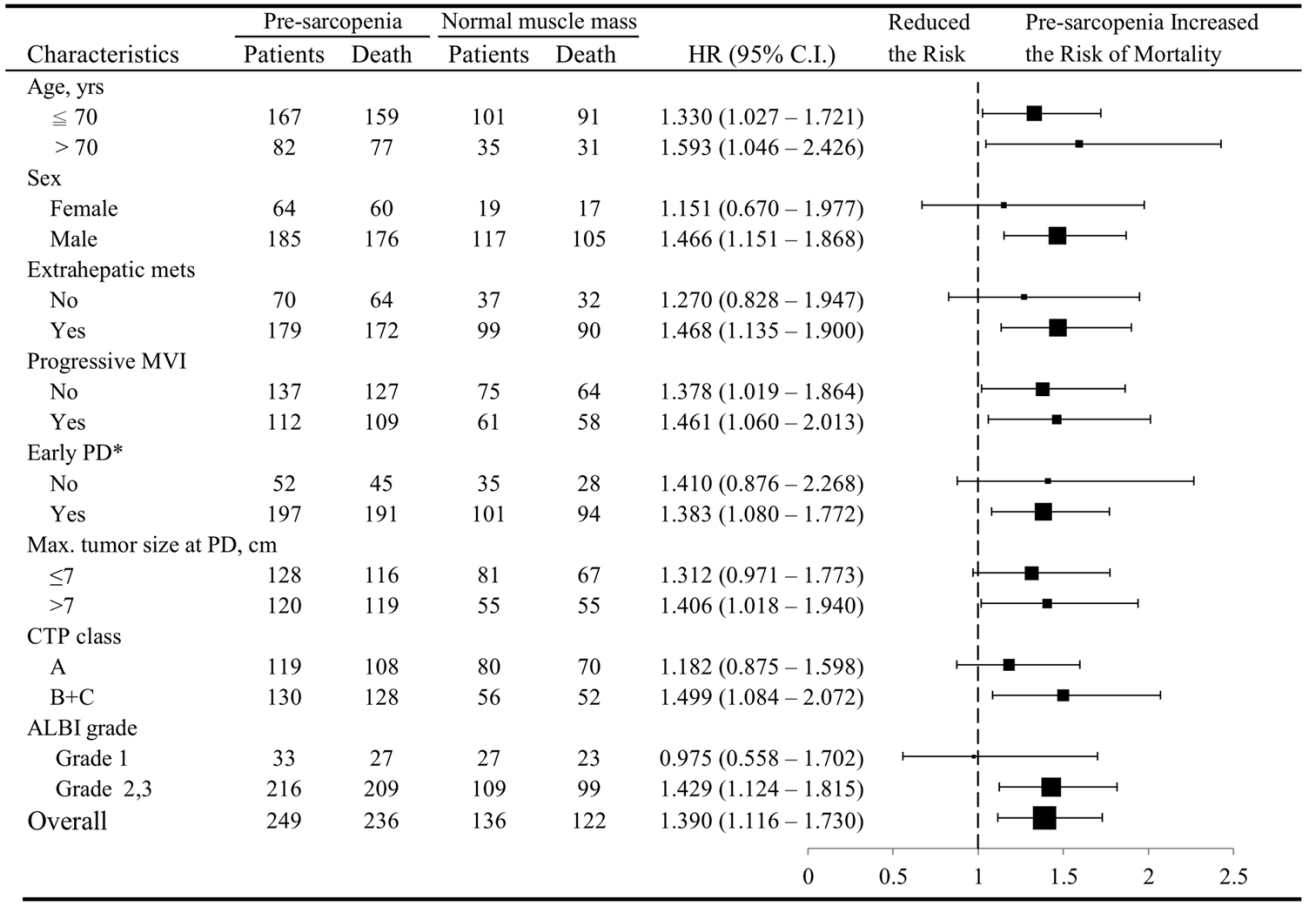
The association between pre-sarcopenia and reduced post-progression survival (PPS) in sorafenib-failed, advanced hepatocellular carcinoma.

The status of muscle depletion was significantly related to worse PPS irrespective of age (Fig. 4). This association was significant in male gender, patients with lower BMI, extrahepatic metastasis, progressive macrovascular invasion, early PD, larger tumor size, and Child–Pugh classes B/C or ALBI grades 2/3.

## Discussion

This is the first study to investigate the role of muscle wasting in sorafenib-failed HCC. Unlike a previous study that enrolled patients at different tumor stage^19^, our patients were homogenous in tumor status. The results of this large cohort study indicated that the presence of pre-sarcopenia was associated with a poor outcome in patients with sorafenib-failed advanced HCC. In addition, we identified older age (> 60 years), female gender, and lower BMI value (< 22 kg/m^2^) while sorafenib failure were independent predictors for the presence of pre-sarcopenia. These findings implied nutrition replacement to maintain muscle mass could prolong survival for higher-risk patients.

Malnutrition is common in patients with cancer or advanced liver disease and could significantly affect their prognosis^11,24^. To evaluate nutrition status in patients with advanced liver disease or cancer, body composition is more important than body weight because the increased weight could be composed of additional water in the form of ascites or edema^24^. Muscle status is a reliable surrogate for nutrition status and physical activity. Unlike ECOG performance status and the subjective classification of ascites and hepatic encephalopathy in the Child–Pugh system, which are traditionally adopted in the assessment and management of HCC, muscle mass status is an objective and quantitative factor to evaluate the patients’ general condition. According to the clinical trials in France and Canada, 27.5–30% of HCC patients had muscle depletion^25,26^. In advanced HCC, the prevalence of muscle wasting was reported as 49% and 65.1% in the Italian and Japanese cohorts, respectively^19,20^. According to previous studies, skeletal muscle depletion did not influence overall survival among patients with early or intermediate-staged HCC^15^. However, it was suggested as an independent predictor of mortality in patients with unresectable HCC, who were undergoing sorafenib therapy^18–22^. In our study with much more patient numbers, the prevalence of pre-sarcopenia (muscle depletion) was 49.6% at the initiation of sorafenib treatment, and increased to 64.7% with the development of progressive disease. Presence of pre-sarcopenia also independently predicted a poor PPS in HCC patients who failed sorafenib treatment. In addition, the survival-predictive role of pre-sarcopenia was more significant in patients with larger tumor size, early PD, extrahepatic metastasis, and progressive vascular invasion of advanced HCC. These findings suggested that muscle depletion deteriorates in line with tumor stage and has to get more serious concern in patients with advanced tumors.

Similar to previous Japanese studies, in which unremarkable association between Child–Pugh class and muscle mass depletion was reported^15,18^, no significant differences in liver function could be observed according to our patients’ muscle status. Muscle wasting was found to predict mortality in cirrhotic patients at Child–Pugh class A and B^27^. In our cohort, the presence of pre-sarcopenia was associated with a significantly worse PPS, particularly in patients at Child–Pugh B or ALBI grade 2 while sorafenib failure. Independent of liver reserves, we still identified older age, female gender, and lower BMI as predictors of pre-sarcopenia in HCC patients who failed sorafenib treatment. The prevalence of muscle depletion was similarly demonstrated to increase with older age and decreased BMI^28,29^. In our cohort, significant reduction of BMI and increased ascites formation were observed in pre-sarcopenic patients. The presence of ascites could overestimate the value of BMI; but it did not affect the risk of muscle loss in patients with BMI ≥ 22 kg/m^2^. In contrast, a significant risk of pre-sarcopenia was identified in patients whose BMI was less than 22 kg/m^2^, even along with the bias of ascites. On the other hand, muscle depletion was more prevalent in female HCC patients in our study. This finding was similar to the results from previous studies^15,20^. Yet, we observed that the association between the presence of pre-sarcopenia and worse PPS was much more significant in the male patients. Female patients, usually with an abundance of adipose tissue, generate energy preferentially from fat that might account for the ostensible resistance to muscle loss compared to the male patients^17,30^.

In our cohort, patients who sustained normal muscle mass from the beginning of sorafenib treatment to treatment failure had better PPS than those who developed pre-sarcopenia. Impressively, the initially pre-sarcopenic patients who increased muscle mass during sorafenib treatment and relieved from pre-sarcopenia were observed to have significantly longer PPS compared with others who kept in muscle depletion. These findings suggested the potential therapeutic strategy of nutrition intervention to prevent muscle wasting and improve clinical outcomes of advanced HCC. In addition to hand-foot syndrome, gastrointestinal adverse events, such as anorexia, vomiting and dyspepsia are also common in sorafenib-treated patients^4,5^. Besides, sorafenib might inhibit carnitine absorption and also lead to pre-sarcopenia^31^. All of these adverse events could exacerbate protein energy malnutrition and associate with poorer survival of HCC patients^16^. Therefore, aggressive nutrition intervention and lifestyle modification are important in patients with sorafenib-treated HCC to improve their prognosis. Several studies have suggested that supplementation of branched-chain amino-acids may be useful in maintaining liver reserves and beneficial to patients treated with sorafenib^32,33^. Exercise therapy might be also promising in preventing skeletal muscle depletion^34^, but further investigations with large cohorts are still needed.

Imaging-based definitions of muscle depletion were diverse and not yet standardized until now. Status of muscle mass could be measured by a cross-sectional area of the psoas muscle^35^, TPMT at the level of the umbilicus normalized by body height^36^, and the third lumbar vertebra muscle index^37,38^. Among these indices, the psoas cross-sectional area and the third lumbar vertebral muscle index could only be measured by the commercialized software with limited accessibility. In contrast, measurement of TPMT is accessible on most CT scan images without special software. According to previous studies^36,39^, muscle depletion defined by the value of TPMT/height less than 16.8 mm/m indicated a higher mortality rate in cirrhotic patients independent of the MELD and MELD-Na scores. This measurement was strongly correlated with the third lumbar vertebral muscle index. In addition to CT scan, measurement of muscle mass from the magnetic resonance imaging (MRI) at the level of the third vertebrae was recently reported to have good survival prediction in decompensated cirrhotic patients^40^. In our study, we measured TPMT/height on CT scan or MRI to evaluate muscle status of our patients. As this measurement could be obtained from radiological images regularly performed for staging purposes or follow-up, it could be promoted in clinical practice to overcome intrinsic limits of bioimpedance and anthropometric measurements, which were hampered by elevated BMI and ascites^20^.

There are several limitations in this study. First, this is a retrospective study that only enrolled patients treated in a single hospital. However, our hospital is the leading tertiary medical center in Taiwan with strict regulations. The information bias would be ameliorated by regular tumor reassessment by radiological images and clinical evaluation. In addition, it is so far the largest real-life Asian cohort of patients with advanced HCC who failed first-line sorafenib treatment. It is also the first study to demonstrate the prognosis-predicting role of pre-sarcopenia in these patients. Second, the muscle strength evaluation, such as hand grip strength and walking speed^12^, which is usually regarded as a diagnostic criterion for sarcopenia was not assessed due to the retrospective design of our study. Third, we could not obtain information about daily calorie intake or nutrition support from our patients. Even dietitian consulting would be applied for all patients at high risk of malnutrition, some of them could restore muscle mass during cancer treatment, but others kept muscle wasting. This finding suggested the diverse implementation rate and effectiveness of nutrition support, and highlighted the unmet need of nutrition intervention in these patients. Fourth, most of our patients had chronic hepatitis B or C. Whether our results could be applied to other patients with underlying alcoholic liver disease is still undetermined.

In conclusion, pre-sarcopenia independently determined the prognosis of sorafenib-failed HCC particularly in patients with intermediate liver reserves. Building muscle mass would be important for patients at higher risk of pre-sarcopenia to improve survival.

## Materials and methods

### Selection of patients

From August 2012 to March 2017, 385 consecutive patients who experienced progressive disease after sorafenib treatment for advanced HCC in Taipei Veterans General Hospital were retrospectively reviewed. All patients were initially diagnosed according to the criteria of American Association for the Study of Liver Diseases (AASLD) treatment guidelines for HCC^41^. According to the strict reimbursement regulations in Taiwan, patients at Barcelona Clinic Liver Cancer (BCLC) stage C and Child–Pugh class A with portal vein invasion or extrahepatic metastasis were approved for sorafenib treatment^9^. PD was defined according to the followed CT scan or MRI which was performed every two months after the start of sorafenib treatment^42^. Patterns of PD were classified as intrahepatic or extrahepatic tumor growth (> 20% increase in tumor size of the preexisting lesions), and new intrahepatic or extrahepatic lesions^43^. Progressive macrovascular invasion was defined as PD in vascular tumor thrombus without newly developed intrahepatic or extrahepatic lesion or progression of existed tumor. This study was conducted in accordance with the Declaration of Helsinki and current ethical guidelines. It was approved by the Institutional Review Board of the Taipei Veterans General Hospital (IRB No.: 2019-07-038BC). Informed consent was waived by the Institutional Review Board of the Taipei Veterans General Hospital due to the retrospective design and most enrolled patients had died.

#### Definition, clinical assessments and outcomes

Muscle mass status was assessed by measuring TPMT on CT scan or MRI at the level of the umbilicus^36,44^. The value of TPMT/BH less than 16.8 mm/m was defined as pre-sarcopenia^36^. In this study, we retrospectively calculated TPMT/BH at the beginning of sorafenib treatment and at tumor PD recognized by image studies. Anthropometric measurements, laboratory exams, including hemogram, serum chemistry, AFP level, as well as Child–Pugh class, ALBI grade, and ECOG performance status were evaluated when PD was confirmed. The PPS was measured from the date of radiology-proven PD to the date of death.

#### Statistical analysis

Continuous variables were expressed as mean ± standard deviation or median (interquartile ranges), while categorical variables were analyzed as frequency and percentages. The Pearson chi-square analysis or Fisher’s exact test was used to compare categorical variables, while the Student t-test or Mann–Whitney U test was applied for continuous variables. The predictive power of a score to predict pre-sarcopenia was assessed using the area under receiver operating characteristic curves (AUROC). Survival was estimated by the Kaplan–Meier method and compared by the log-rank test. Additionally, Cox’s proportional-hazard model was used to identify prognostic factors of survival. For all analyses, *p* < 0.05 was considered statistically significant. All statistical analyses were performed using the Statistical Package for Social Sciences (SPSS Statistics v. 17.0 for Windows, Armonk, NY, USA).

## Supplementary information

Supplementary Figure 1.

## Data Availability

The datasets generated during and analyzed during the current study are available from the corresponding author on reasonable request.

## Abbreviations

AE: Adverse events
AFP: Alpha-fetoprotein
ALBI grade: Albumin-bilirubin grade
ALT: Alanine aminotransferase
AST: Aspartate aminotransferase
BCLC: Barcelona-Clinic-Liver-Cancer
CI: Confidence interval
CT: Computed tomography
HBV: Hepatitis B virus
HCC: Hepatocellular carcinoma
HCV: Hepatitis C virus
HR: Hazard ratio
ICI: Immune checkpoint inhibitors
INR: International normalized ratio
LD: Liver decompensation
Mets: Metastasis
mRECIST: Modified Response Evaluation Criteria in Solid Tumors
MRI: Magnetic resonance image
MVI: Macroscopic vascular invasion
NA: Not adopted
NEH: New extrahepatic metastasis
NS: Not significant
OS: Overall survival
PD: Progressive disease
PFS: Progression-free survival
PPS: Post-progression survival
