# Supplementary material for: Pre-sarcopenia determines post-progression outcomes in advanced hepatocellular carcinoma after sorafenib failure
Source: Sci Rep. 2020 Oct 27;10:18375. doi: 10.1038/s41598-020-75198-z (PMC7591538; doi:10.1038/s41598-020-75198-z)
Supplement: Supplementary file 1 — Supplementary Figure 1. [file 41598_2020_75198_MOESM1_ESM.docx]

Supplementary Figure 1**. Subgroup analysis of post-progression survival (PPS) according the development or resolution of pre-sarcopenia during sorafenib treatment**


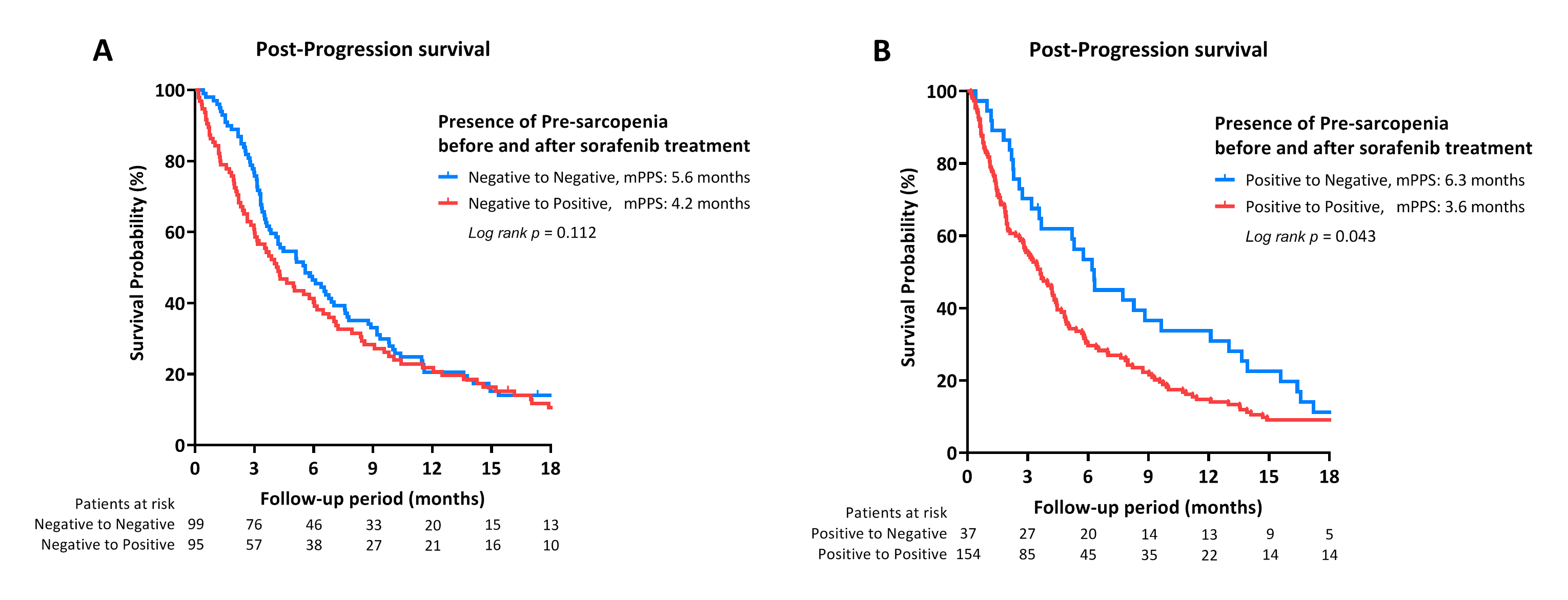


Subgroup analysis of PPS based on the development (A) and resolution (B) of pre-sarcopenia during sorafenib treatment.
